# Supplementary material for: Chromatin protein complexes involved in gene repression in lamina-associated domains
Source: EMBO J. 2024 Sep 25;43(21):5260–87. doi: 10.1038/s44318-024-00214-1 (PMC11535540; doi:10.1038/s44318-024-00214-1)
Supplement: Supplementary file 1 — Appendix [file 44318_2024_214_MOESM1_ESM.pdf]

Chromatin protein complexes involved in gene repression in lamina-associated domains

Stefano G Manzo, Abdelghani Mazouzi, Christ Leemans, Tom van Schaik, Nadia Neyazi, Marjon S van Ruiten, Benjamin D Rowland, Thijn Brummelkamp, Bas van Steensel

APPENDIX

TABLE OF CONTENTS

- **Appendix Figure S1... 2**
- **Appendix Figure S2... 3**
- **Appendix Figure S3... 4**
- **Appendix Table S1...5**

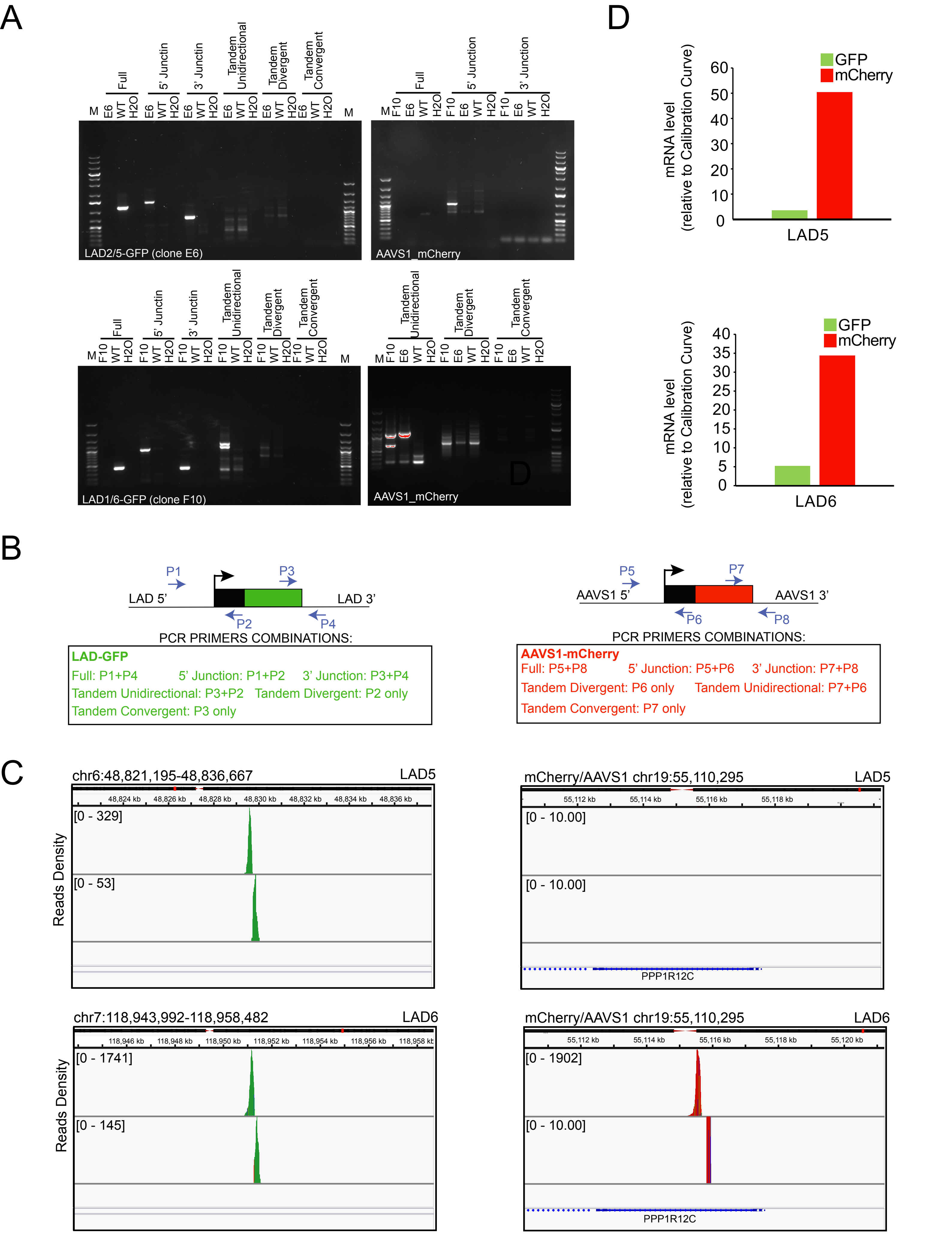

Appendix Figure S1 A) Genotyping PCR for GFP and mCherry reporters in the selected genomic locations. Genomic DNA from wild-type HAP1 cells was used as negative control. B) Schematic of all primer combinations used in the genotyping PCR. C) Tag-Map reads around integration sites for LAD-GFP reporters and AAVS1-mCherry. D) mRNA level for GFP and mCherry for the two LAD-reporter cell lines. mRNA levels were calculated by interpolating qPCR CT values on a calibration curve generated with GFP and mCherry plasmids to intrinsically normalize for primer efficiency.

A

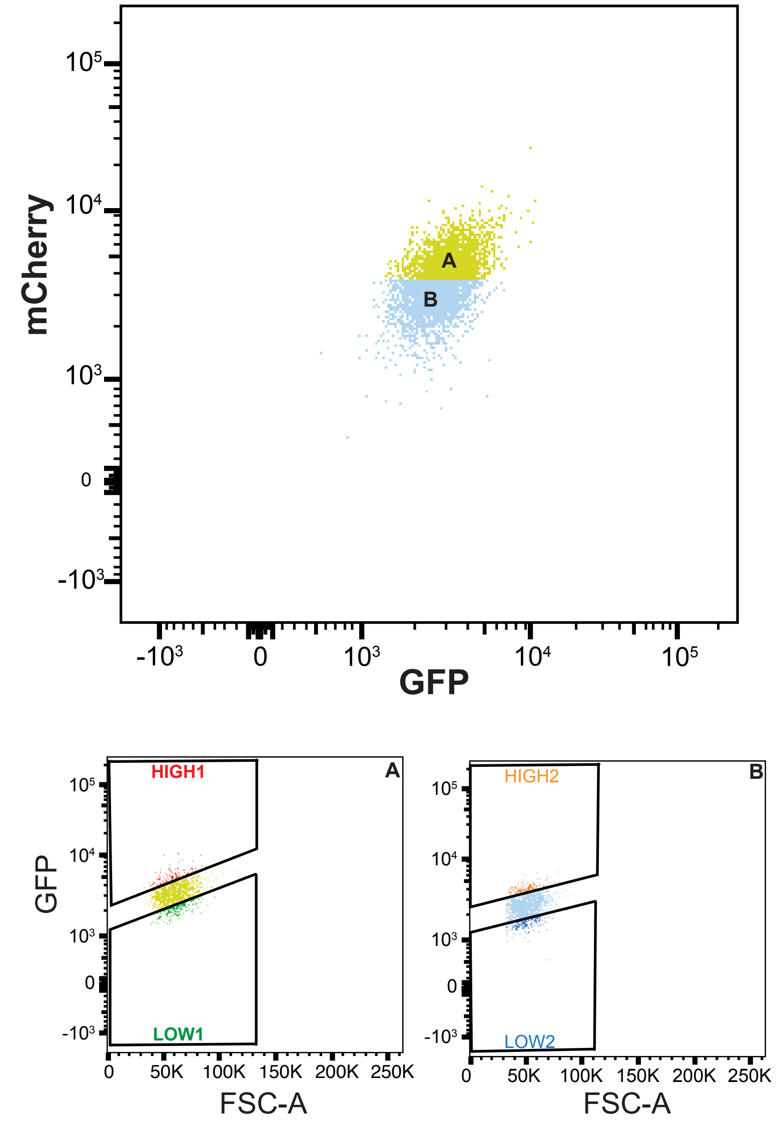

B

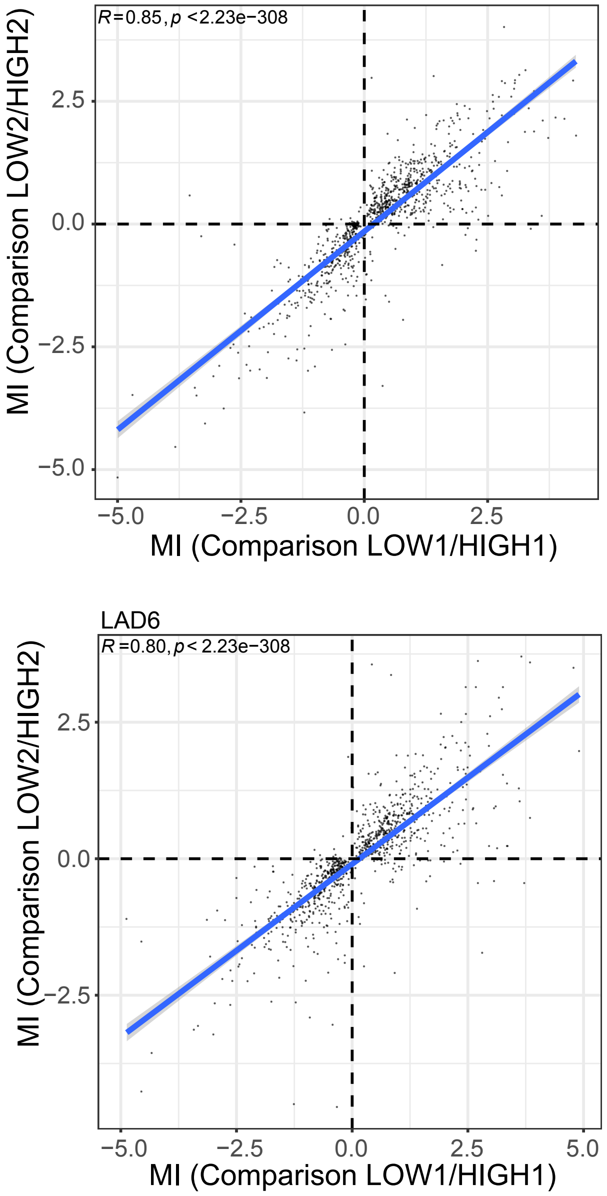

C

LAD5 G:Profiler analysis

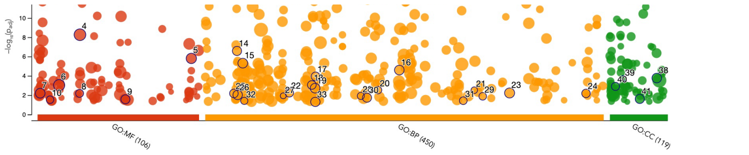

ID	Source	Term ID	Term Name	p_{adj} (query_1)
1	GO:MF	GO:0005515	protein binding	3.188×10^{-43}
2	GO:MF	GO:0003712	transcription coregulator activity	8.332×10^{-40}
3	GO:MF	GO:0140993	histone modifying activity	2.760×10^{-17}
4	GO:MF	GO:0016740	transferase activity	5.489×10^{-9}
5	GO:MF	GO:0140640	catalytic activity, acting on a nucleic acid	1.565×10^{-6}
6	GO:MF	GO:0005524	ATP binding	9.335×10^{-4}
7	GO:MF	GO:0001227	DNA-binding transcription repressor activity, R...	6.290×10^{-3}
8	GO:MF	GO:0016706	2-oxoglutarate-dependent dioxygenase activity	6.549×10^{-3}
9	GO:MF	GO:0045182	translation regulator activity	2.684×10^{-2}
10	GO:MF	GO:0004532	RNA exonuclease activity	2.956×10^{-2}
11	GO:BP	GO:0044271	cellular nitrogen compound biosynthetic process	1.391×10^{-97}
12	GO:BP	GO:0043484	regulation of RNA splicing	4.866×10^{-19}
13	GO:BP	GO:0043632	modification-dependent macromolecule catab...	1.746×10^{-15}
14	GO:BP	GO:0006403	RNA localization	2.625×10^{-7}
15	GO:BP	GO:0006913	nucleocytoplasmic transport	4.860×10^{-6}
16	GO:BP	GO:0048511	rhythmic process	2.529×10^{-5}
17	GO:BP	GO:0031929	TOR signaling	1.144×10^{-4}
18	GO:BP	GO:0031123	RNA 3'-end processing	8.865×10^{-4}
19	GO:BP	GO:0031647	regulation of protein stability	1.905×10^{-3}
20	GO:BP	GO:0045324	late endosome to vacuole transport	2.996×10^{-3}
21	GO:BP	GO:0080009	mRNA methylation	3.067×10^{-3}
22	GO:BP	GO:0019080	viral gene expression	5.758×10^{-3}
23	GO:BP	GO:0198738	cell-cell signaling by wnt	5.968×10^{-3}
24	GO:BP	GO:1990830	cellular response to leukemia inhibitory factor	6.579×10^{-3}
25	GO:BP	GO:0006090	pyruvate metabolic process	6.652×10^{-3}
26	GO:BP	GO:0006470	protein dephosphorylation	9.423×10^{-3}
27	GO:BP	GO:0017196	N-terminal peptidyl-methionine acetylation	1.176×10^{-2}
28	GO:BP	GO:0042659	regulation of cell fate specification	1.201×10^{-2}
29	GO:BP	GO:0090503	RNA phosphodiester bond hydrolysis, exonucle...	1.326×10^{-2}
30	GO:BP	GO:0043433	negative regulation of DNA-binding transcripti...	1.858×10^{-2}
31	GO:BP	GO:0071985	multivesicular body sorting pathway	3.773×10^{-2}
32	GO:BP	GO:0007064	mitotic sister chromatid cohesion	3.903×10^{-2}
33	GO:BP	GO:0031669	cellular response to nutrient levels	4.997×10^{-2}
34	GO:CC	GO:0005654	nucleoplasm	1.540×10^{-129}
35	GO:CC	GO:1902493	acetyltransferase complex	3.269×10^{-24}
36	GO:CC	GO:0016592	mediator complex	1.473×10^{-13}
37	GO:CC	GO:0036464	cytoplasmic ribonucleoprotein granule	2.651×10^{-13}
38	GO:CC	GO:0098798	mitochondrial protein-containing complex	1.702×10^{-4}
39	GO:CC	GO:0030289	protein phosphatase 4 complex	1.802×10^{-4}
40	GO:CC	GO:0005844	polysome	1.242×10^{-3}
41	GO:CC	GO:0044391	ribosomal subunit	2.481×10^{-2}

LAD6 G:Profiler analysis

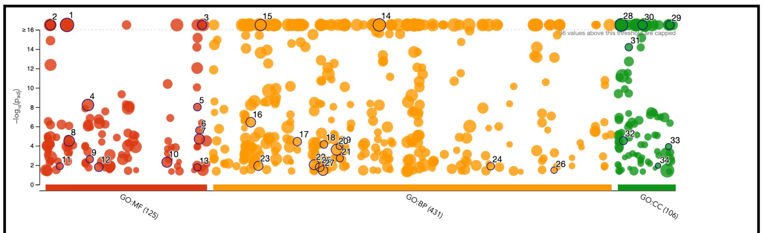

ID	Source	Term ID	Term Name	p_{adj} (query_1)
1	GO:MF	GO:0005515	protein binding	2.590×10^{-47}
2	GO:MF	GO:0003712	transcription coregulator activity	7.819×10^{-31}
3	GO:MF	GO:0140993	histone modifying activity	4.620×10^{-20}
4	GO:MF	GO:0016740	transferase activity	6.013×10^{-9}
5	GO:MF	GO:0140223	general transcription initiation factor activity	9.846×10^{-9}
6	GO:MF	GO:0140658	ATP-dependent chromatin remodeler activity	2.500×10^{-6}
7	GO:MF	GO:0140640	catalytic activity, acting on a nucleic acid	1.964×10^{-5}
8	GO:MF	GO:0008270	zinc ion binding	3.058×10^{-5}
9	GO:MF	GO:0016896	RNA exonuclease activity, producing 5'-phosp...	2.425×10^{-3}
10	GO:MF	GO:0060090	molecular adaptor activity	4.959×10^{-3}
11	GO:MF	GO:0004693	cyclin-dependent protein serine/threonine kina...	1.287×10^{-2}
12	GO:MF	GO:0019783	ubiquitin-like protein peptidase activity	1.599×10^{-2}
13	GO:MF	GO:0140142	nucleocytoplasmic carrier activity	1.690×10^{-2}
14	GO:BP	GO:0044271	cellular nitrogen compound biosynthetic process	7.041×10^{-106}
15	GO:BP	GO:0009057	macromolecule catabolic process	7.337×10^{-28}
16	GO:BP	GO:0006913	nucleocytoplasmic transport	3.656×10^{-7}
17	GO:BP	GO:0019080	viral gene expression	3.677×10^{-5}
18	GO:BP	GO:0032008	positive regulation of TOR signaling	7.185×10^{-5}
19	GO:BP	GO:0033962	P-body assembly	1.073×10^{-4}
20	GO:BP	GO:0033365	protein localization to organelle	2.531×10^{-4}
21	GO:BP	GO:0034063	stress granule assembly	1.959×10^{-3}
22	GO:BP	GO:0030098	lymphocyte differentiation	9.024×10^{-3}
23	GO:BP	GO:0007623	circadian rhythm	1.202×10^{-2}
24	GO:BP	GO:0090503	RNA phosphodiester bond hydrolysis, exonucle...	1.285×10^{-2}
25	GO:BP	GO:0031123	RNA 3'-end processing	1.625×10^{-2}
26	GO:BP	GO:1903025	regulation of RNA polymerase II regulatory regi...	3.325×10^{-2}
27	GO:BP	GO:0031647	regulation of protein stability	3.688×10^{-2}
28	GO:CC	GO:0005654	nucleoplasm	1.368×10^{-135}
29	GO:CC	GO:1902493	acetyltransferase complex	1.074×10^{-19}
30	GO:CC	GO:0036464	cytoplasmic ribonucleoprotein granule	3.503×10^{-17}
31	GO:CC	GO:0016592	mediator complex	6.337×10^{-15}
32	GO:CC	GO:0005844	polysome	2.944×10^{-5}
33	GO:CC	GO:0140288	GBAF complex	1.221×10^{-4}
34	GO:CC	GO:0071986	Ragulator complex	1.132×10^{-2}

Appendix Figure S2. A) Sorting strategy for the screen. Gene-trapped cells were divided in two bins according to mCherry fluorescent intensity (subgroup A and B, top panel). Each subgroup was further sorted according to GFP intensity (HIGH vs LOW, bottom panels). FSC-A is forward scattering area used for gating. B) Correlation plot between mutation indexes for significant hits (fcpv < 0.05) for LOW1/HIGH1 comparison vs LOW2/HIGH2 comparison (see also figure 1C) for GFP reporters. The blue line represents a linear model, Pearson correlation and P values are shown in the plot. B) G:Profiler analysis (Kolberg et al., 2023) for regulators of GFP reporters for LAD5 and LAD6 cell lines.

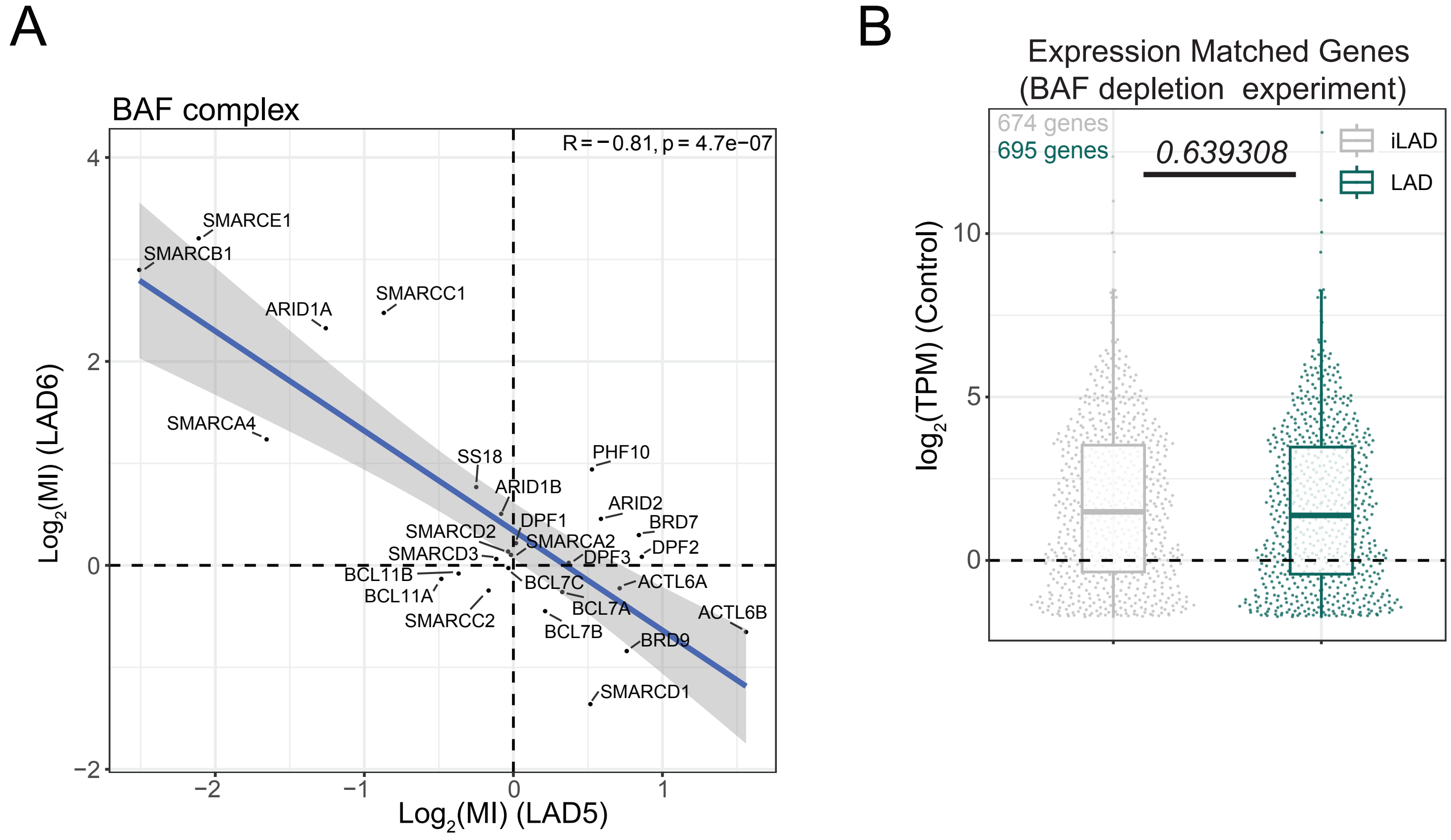

Appendix Figure S3 A) Correlation of LAD5 and LAD6 MIs from the screen for all subunits composing the BAF complex (cBAF and pBAF). The blue line represents a fitted linear model; Pearson correlation and P values are shown in the plots B) Gene expression levels for expression-matched LAD and iLAD genes in the BAF depletion experiments. Data are from Schick et al. 2019, and are the results from three biological replicates.

A

Jaeger et al Nat gen 2020

degron	MED1	MED6	MED10	MED12	MED14	MED26	MED28	MED31
up	0	78	12	0	67	0	0	0
down	3232	0	70	1	3046	0	6	0

Appendix Table S1. A) Number of differentially expressed genes following acute depletion of 8 different Mediator subunits (Jaeger et al., 2020)
