# Supplementary material for: Chromatin protein complexes involved in gene repression in lamina-associated domains
Source: EMBO J. 2024 Sep 25;43(21):5260–87. doi: 10.1038/s44318-024-00214-1 (PMC11535540; doi:10.1038/s44318-024-00214-1)
Supplement: Supplementary file 5 — Expanded View Figures [file 44318_2024_214_MOESM5_ESM.pdf]

Expanded View Figures

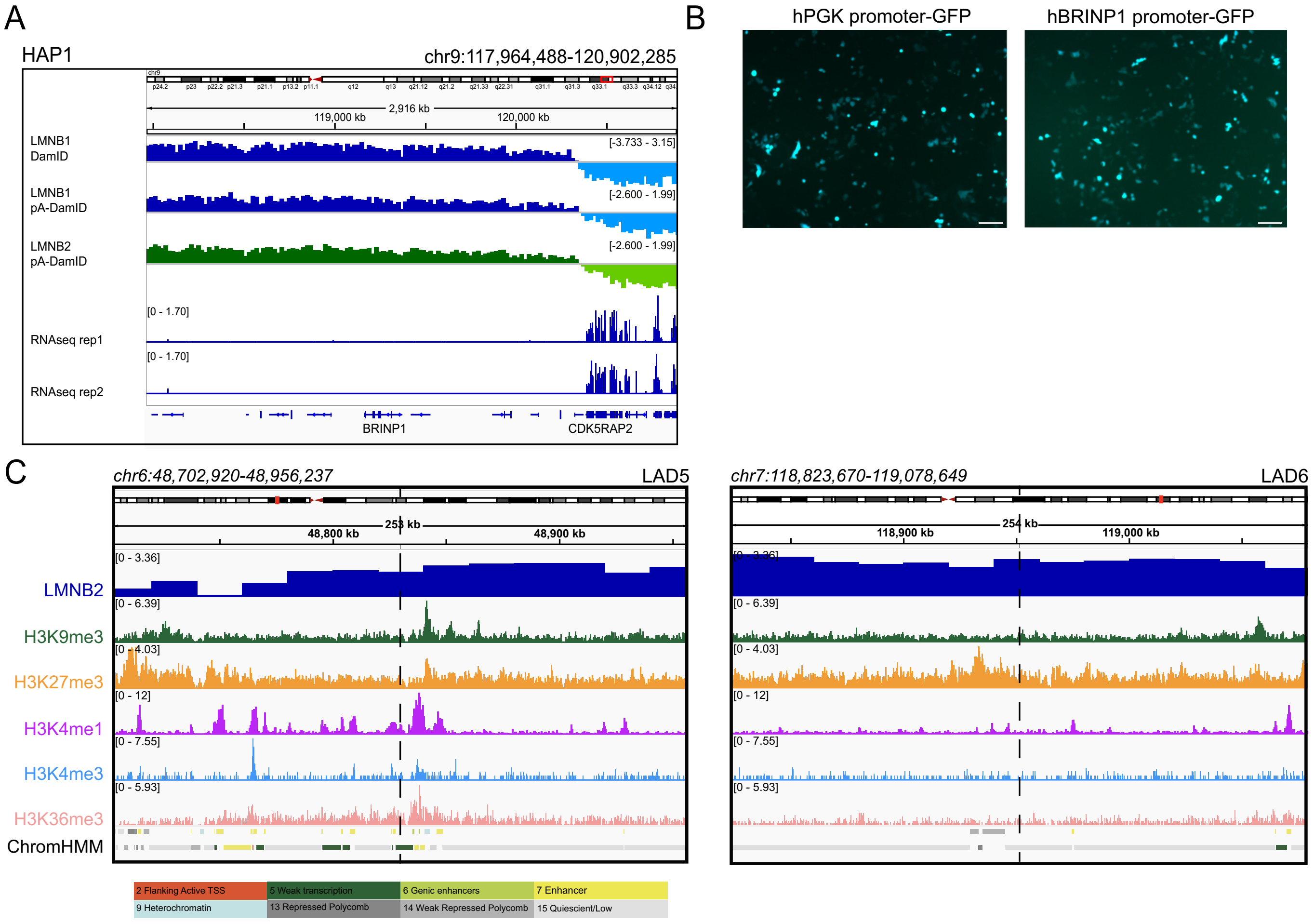

Figure EV1. Characterization of BRINP1 promoter and the chromatin environment around LAD reporters' integration sites in HAP1 cells.

(**A**) Top tracks: IGV tracks showing DNA-NL contacts (generated by in vivo DamID, LMNB1 pA-DamID, and LMNB2 pA-DamID) of 3 Mb region surrounding the BRINP1 gene. Bottom tracks: RNA-seq levels for *BRINP1* and surrounding genes in the same 3 Mb genomic regions. (**B**) Transfection of HAP1 cells with *phPGK::GFP* or *pBRINP1::GFP* plasmids. *BRINP1* promoter is active in HAP1 cells when expressed in an episomal setting. Scale is at 20 μm. (**C**) DamID tracks for LMNB2 and ChIP-seq data for H3K9me3, H3K27me3, H3K4me1, H3K4me3, and H3K36me3 in HAP1 in a 250 kb window surrounding LAD5 and LAD6 reporter integrations (dashed lines). Data are from (Haarhuis et al, 2022). The bottom track shows annotations of major chromatin states according to ChromHMM (Ernst and Kellis, 2012); corresponding color key is shown in the bottom panel. Results are from at least two biological replicates.

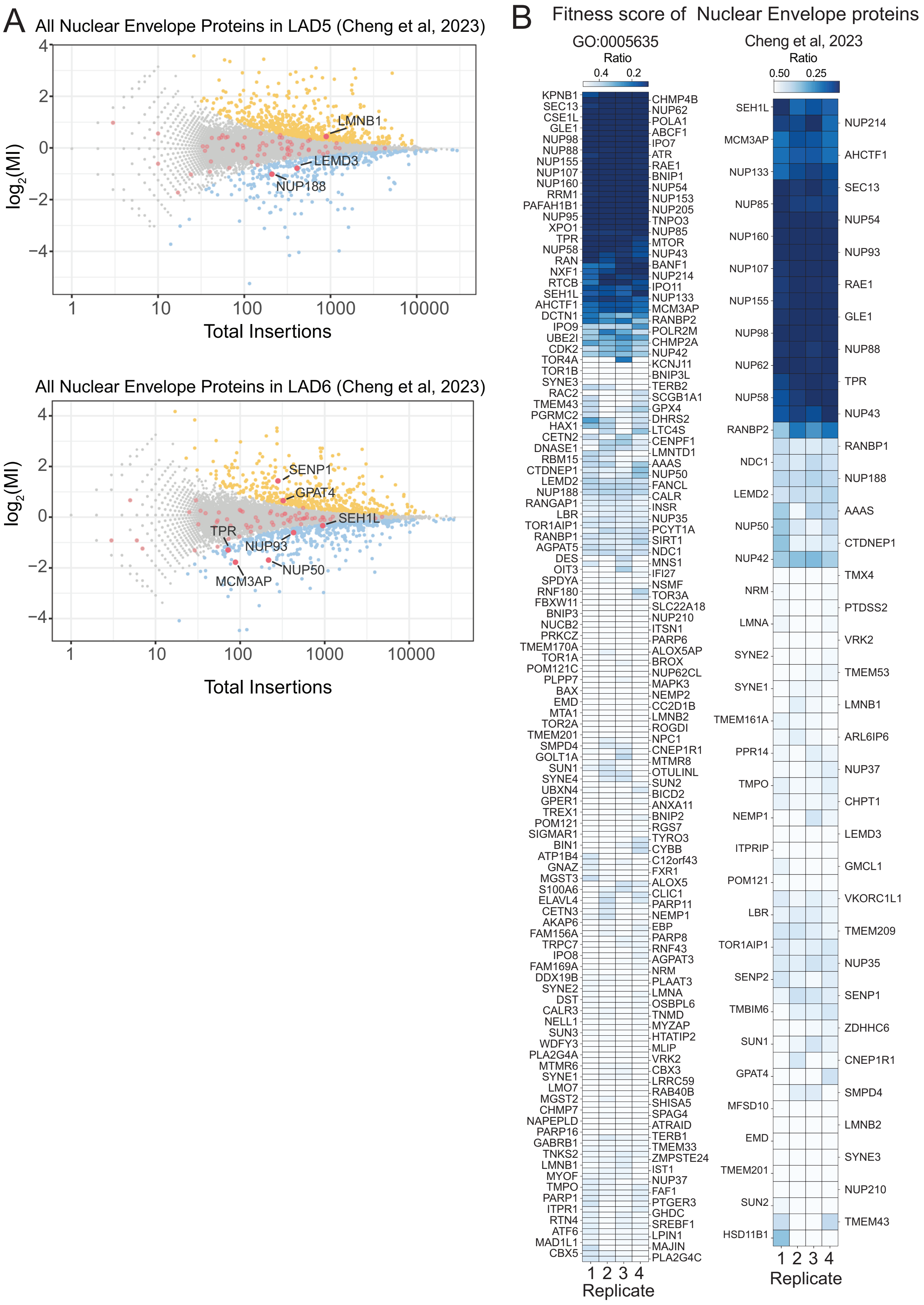
A
All Nuclear Envelope Proteins in LAD5 (Cheng et al, 2023)
log2(MI)
Total Insertions
1
10
100
1000
10000
4
2
0
−2
−4
LMNB1
LEMD3
NUP188
All Nuclear Envelope Proteins in LAD6 (Cheng et al, 2023)
SENP1
GPAT4
SEH1L
TPR
NUP93
NUP50
MCM3AP
B
Fitness score of Nuclear Envelope proteins
GO:0005635
Ratio
0.4
0.2
KPNB1 SEC13 CSE1L GLE1 NUP98 NUP88 NUP155 NUP107 NUP160 RRM1 PAFAH1B1 NUP95 XPO1 TPR NUP58 RAN NXF1 RTCB SEH1L AHCTF1 DCTN1 IPO9 UBE2I CDK2 TOR4A TOR1B SYNE3 RAC2 TMEM43 PGRMC2 HAX1 CETN2 DNASE1 RBM15 CTDNEP1 LEMD2 NUP188 RANGAP1 LBR TOR1AIP1 RANBP1 AGPAT5 DES OIT3 SPDYA RNF180 FBXW11 BNIP3 NUCB2 PRKCZ TMEM170A TOR1A POM121C PLPP7 BAX EMD MTA1 TOR2A TMEM201 SMPD4 GOLT1A SUN1 SYNE4 UBXN4 GPER1 TREX1 POM121 SIGMAR1 BIN1 ATP1B4 GNAZ MGST3 S100A6 ELAVL4 CETN3 AKAP6 FAM156A TRPC7 IPO8 FAM169A DDX19B SYNE2 DST CALR3 NELL1 SUN3 WDFY3 PLA2G4A MTMR6 SYNE1 LMO7 MGST2 CHMP7 NAPEPLD PARP16 GABRB1 TNKS2 LMNB1 MYOF TMPO PARP1 ITPR1 RTN4 ATF6 MAD1L1 CBX5
CHMP4B NUP62 POLA1 ABCF1 IPO7 ATR RAE1 BNIP1 NUP54 NUP153 NUP205 TNPO3 NUP85 MTOR NUP43 BANF1 NUP214 IPO11 NUP133 MCM3AP RANBP2 POLR2M CHMP2A NUP42 KCNJ11 BNIP3L TERB2 SCGB1A1 GPX4 DHRS2 LTC4S CENPF1 LMNTD1 AAAS NUP50 FANCL CALR INSR NUP35 PCYT1A SIRT1 NDC1 MNS1 IFI27 NSMF TOR3A SLC22A18 NUP210 ITSN1 PARP6 ALOX5AP BROX NUP62CL MAPK3 NEMP2 CC2D1B LMNB2 ROGDI NPC1 CNEP1R1 MTMR8 OTULINL SUN2 BICD2 ANXA11 BNIP2 RGS7 TYRO3 CYBB C12orf43 FXR1 ALOX5 CLIC1 PARP11 NEMP1 EBP PARP8 RNF43 AGPAT3 NRM PLAAT3 LMNA OSBPL6 TNMD MYZAP HTATIP2 MLIP VRK2 CBX3 LRRC59 RAB40B SHISA5 SPAG4 ATRAID TERB1 TMEM33 ZMPSTE24 IST1 NUP37 FAF1 PTGER3 GHDC SREBF1 LPIN1 MAJIN PLA2G4C
1 2 3 4
Replicate
Cheng et al, 2023
Ratio
0.50
0.25
SEH1L MCM3AP NUP133 NUP85 NUP160 NUP107 NUP155 NUP98 NUP62 NUP58 RANBP2 NDC1 LEMD2 NUP50 NUP42 NRM LMNA SYNE2 SYNE1 TMEM161A PPR14 TMPO NEMP1 ITPRIP POM121 LBR TOR1AIP1 SENP2 TMBIM6 SUN1 GPAT4 MFSD10 EMD TMEM201 SUN2 HSD11B1
NUP214 AHCTF1 SEC13 NUP54 NUP93 RAE1 GLE1 NUP88 TPR NUP43 RANBP1 NUP188 AAAS CTDNEP1 TMX4 PTDSS2 VRK2 TMEM53 LMNB1 ARL6IP6 NUP37 CHPT1 LEMD3 GMCL1 VKORC1L1 TMEM209 NUP35 SENP1 ZDHHC6 CNEP1R1 SMPD4 LMNB2 SYNE3 NUP210 TMEM43
1 2 3 4
Replicate

© The Author(s)

Figure EV2. Nuclear Lamina genes in the screen and their essentiality in HAP1 cells.

(**A**) Biochemically identified NE proteins (Cheng et al, 2019) (red dots) highlighted in the screen fishtail plots for LAD5 (top) and LAD6 (bottom). The majority of these NE proteins are not significant hits in the screens. Names of the handful of significant screen hits are indicated in black. (**B**) Essentiality scores of proteins in GO category GO:0005635 (left panel) and of proteins biochemically identified as NE proteins (Cheng et al, 2019), (right panel). Heatmaps show the ratio of sense insertions to the total insertions in wild-type HAP1 cells across 4 independent replicates under untreated conditions. Data are from (Blomen et al, 2015). The scores represent the ratio of disruptive insertions (sense) to the total insertions (sense "disruptive" + antisense "non-disruptive") within the intronic regions of each gene. Genes crucial for cell viability will have fewer disruptive insertions as these cells are depleted, whereas cells with non-disruptive (antisense) insertions survive. As disruptive and non-disruptive integrations occur at similar frequencies, the ratio of insertions in the surviving population indicates whether a gene is important for cell fitness (Blomen et al, 2015). The lower the ratio (blue shading), the more important the gene is for HAP1 cell fitness. Results from 4 different biological replicates are shown separately.

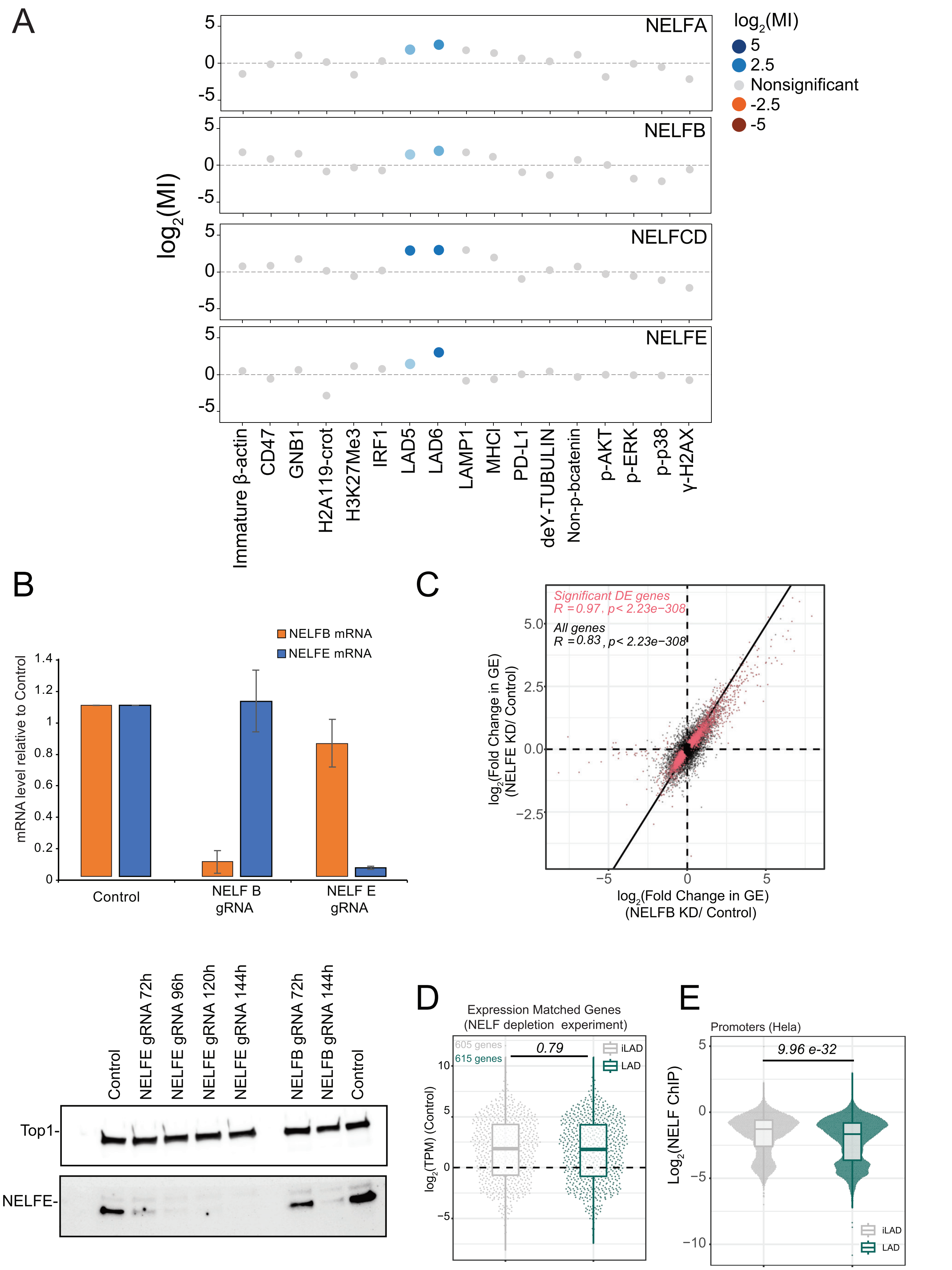
A
log2(MI)
NELFA
NELFB
NELFCD
NELFE
5
2.5
Nonsignificant
-2.5
-5
Immature β-actin
CD47
GNB1
H2A119-crot
H3K27Me3
IRF1
LAD5
LAD6
LAMP1
MHCI
PD-L1
deY-TUBULIN
Non-p-bcatenin
p-AKT
p-ERK
p-p38
γ-H2AX
B
NELFB mRNA
NELFE mRNA
mRNA level relative to Control
Control
NELF B gRNA
NELF E gRNA
C
Significant DE genes
R = 0.97, p < 2.23e−308
All genes
R = 0.83, p < 2.23e−308
log2(Fold Change in GE) (NELFE KD/ Control)
log2(Fold Change in GE) (NELFB KD/ Control)
Control
NELFE gRNA 72h
NELFE gRNA 96h
NELFE gRNA 120h
NELFE gRNA 144h
NELFB gRNA 72h
NELFB gRNA 144h
Control
Top1-
NELFE-
D
Expression Matched Genes
(NELF depletion experiment)
605 genes
615 genes
0.79
iLAD
LAD
log2(TPM) (Control)
E
Promoters (Hela)
9.96 e-32
Log2(NELF ChIP)
iLAD
LAD

 © The Author(s)

Figure EV3. NELF depletion in HAP1.

(**A**) The mutational index (MI) for subunits from the NELF complex (NELFA, NEFLB, NELFCD, NELFE) was plotted for the current LAD5 and LAD6 screens and 15 additional FACS-based haploid screens previously conducted in HAP1 cells using the indicated readouts (blue, negative regulator; orange, positive regulator; gray, not significant) (Brockmann et al, 2017; Haahr et al, 2022; Jongsma et al, 2021; Logtenberg et al, 2019; Mazouzi et al, 2023; Mezzadra et al, 2017; Nieuwenhuis et al, 2017). (**B**) NELF depletion by CRISPRi in HAP1. Top panel: mRNA levels (measured by RT-qPCR) for *NELFE* and *NELFB* following CRISPRi depletion using *NELFE* and *NELFB* specific guide RNAs, 144 h after transduction. Data were first normalized on *GAPDH* mRNA and then on *NELFE/B* mRNA levels in control cells. The error bar represents standard deviation. Results were from three replicates. Bottom panel: detection of NELFE protein levels following CRISPRi depletion using *NELFE* and *NELFB* specific guide RNAs at different timepoints after transduction. DNA topoisomerase 1 (Top1) antibody was used as loading control. (**C**) Correlation between changes in gene expression following NELFB and NELFE knockdowns, for all (black) and significantly de-regulated genes (red). Results were from three replicates for NELFE depletion and two replicates for NELFB depletion. The black line is the diagonal. (**D**) Gene expression levels for expression-matched LAD and iLAD genes in the NELF depletion experiment. (**E**) NELFE levels at promoters of genes in LADs and expression-matched genes in iLADs in HeLa cells. ChIP-seq data are from (Beckedorff et al, 2020). Results are from two biological replicates. *P* value is according to Wilcoxon's test. For (**D**, **E**), the central line in the boxplots represents the median. The lower and upper hinges correspond to the first and third quartiles (the 25th and 75th percentiles). The upper and lower whiskers extends from the hinge to the largest or smallest values respectively no further than 1.5 * inter-quartile range. Outliers were removed only for visualization purposes. Source data are available online for this figure.

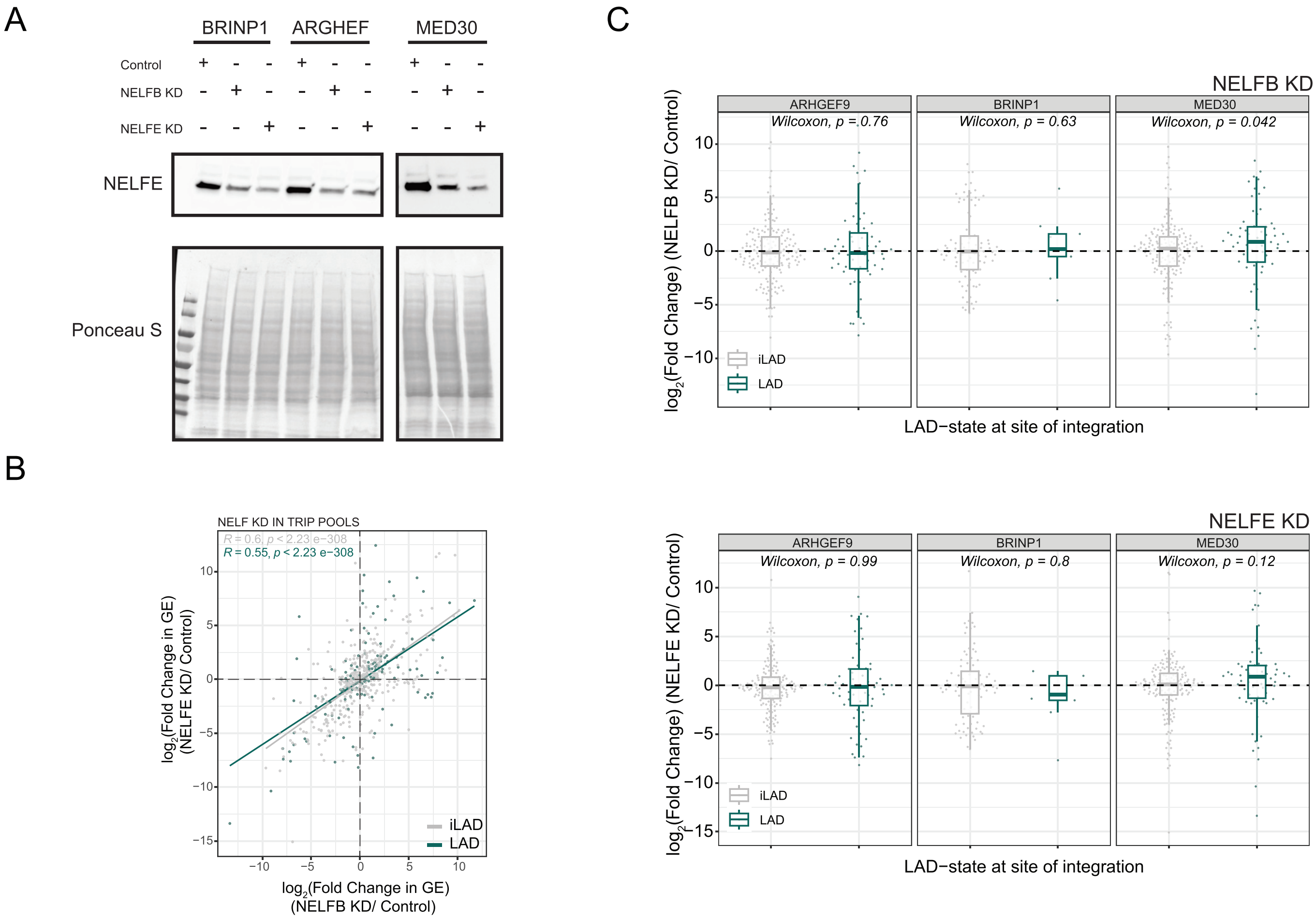

Figure EV4. NELF depletion in K562 TRIP pools.

(**A–C**) Multiplexed detection of the effect of depletion of NELFB and NELFE on the expression levels of reporter genes randomly integrated throughout the genome in K562 cell pools. Barcoded reporters were driven by promoters from the *ARHGEF9*, *BRINP1*, and *MED30* genes as indicated. Cell pools are from (Leemans et al, 2019). (**A**) Western blot of NELFE showing partial knockdown after siRNA-mediated depletion of NELFB or NELFE. (**B**) Changes in expression of the reporters for the three promoters throughout the genome correlate between NELFB and NELFE knockdowns. Reporters integrated in LADs are shown in green, reporters in iLADs are shown in gray. The gray and green lines represent a fitted linear model for iLAD and LAD integrations, respectively; Pearson correlation and *P* values are shown in the plots. (**C**) Changes in expression of each reporter (log_2 scale) after siRNA-mediated knock-down of NELFB (top panel) and NELFE (bottom panel), divided by location in either LADs or iLADs. Results are from three replicates for P_{BRINP1} and two replicates for P_{MED30} and $P_{ARHGEF9}$. *P*-values comparing the distributions in LADs and iLADs are according to Wilcoxon test. Source data are available online for this figure.

 © The Author(s)

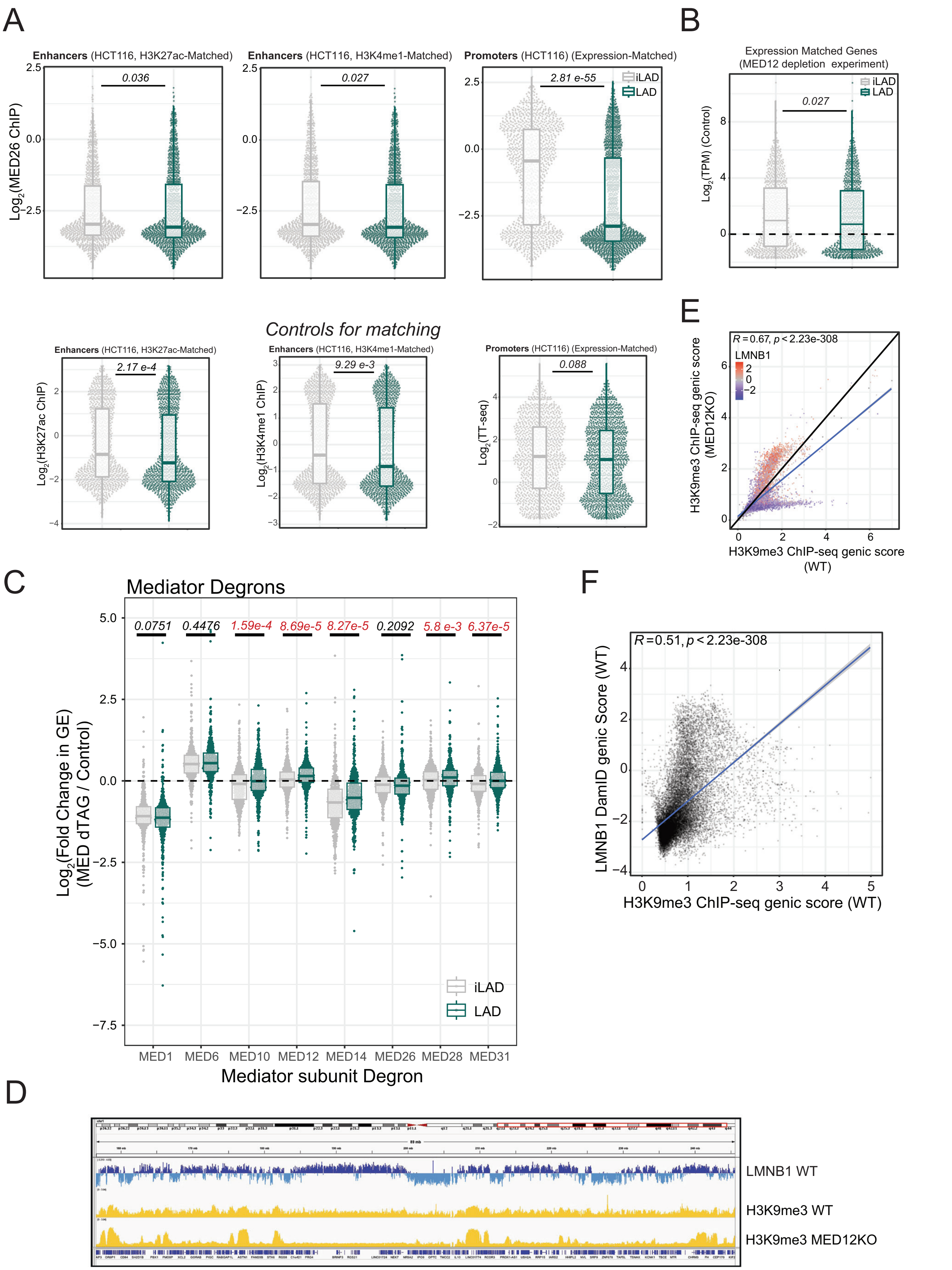
A
Enhancers (HCT116, H3K27ac-Matched)
Enhancers (HCT116, H3K4me1-Matched)
Promoters (HCT116) (Expression-Matched)
0.036
0.027
2.81 e-55
iLAD
LAD
Log2(MED26 ChIP)
Controls for matching
2.17 e-4
9.29 e-3
0.088
Log2(H3K27ac ChIP)
Log2(H3K4me1 ChIP)
Log2(TT-seq)
B
Expression Matched Genes
(MED12 depletion experiment)
0.027
Log2(TPM) (Control)
E
R = 0.67, p < 2.23e-308
LMNB1
H3K9me3 ChIP-seq genic score (MED12KO)
H3K9me3 ChIP-seq genic score (WT)
C
Mediator Degrons
0.0751 0.4476 1.59e-4 8.69e-5 8.27e-5 0.2092 5.8 e-3 6.37e-5
Log2(Fold Change in GE) (MED dTAG / Control)
MED1 MED6 MED10 MED12 MED14 MED26 MED28 MED31
Mediator subunit Degron
F
R = 0.51, p < 2.23e-308
LMNB1 DamID genic Score (WT)
H3K9me3 ChIP-seq genic score (WT)
D
LMNB1 WT
H3K9me3 WT
H3K9me3 MED12KO

Figure EV5. Characterization of Mediator complex in LADs.

(**A**) Top panels: MED26 binding at promoters and enhancers in HCT116 cells. Promoters were matched for TT-seq level to compare LAD and iLAD genes with similar transcriptional activity. Enhancers were matched for H3K27ac or H3K4me1 levels to compare LAD and iLAD regulatory elements with similar enhancer activity. *P* values in are according to Wilcoxon's test. Bottom panels: plot showing the similar distributions of H3K27ac (left) or H3K4me1 (middle) for matched sets of enhancers; and TT-seq levels for matched sets of promoters (right) in LADs and iLADs.Data are from two biological replicates. (**B**) Gene expression levels for expression-matched LAD and iLAD genes in the MED12 depletion experiments. Data are from (Haarhuis et al, 2022) and results are from 6 biological replicates. (**C**) Log_2(fold change) in gene expression (GE) following acute depletion of Mediator subunits for expression-matched LAD and iLAD genes. Statistical significance was calculated with Wilcoxon test for comparison of median and significant *P* values are highlighted in red. Results are from three biological replicates (**D**) IGV genomic tracks for 89 Mb of Chromosome 1 showing LMNB1 DamID profile for HAP1 WT (blue) and H3K9me3 ChIP-seq scores for HAP1 WT and MED12 KO (yellow). The genomic tracks show increased compartmentalization of heterochromatin in LADs following MED12 knockout. (**E**) Correlation between H3K9me3 levels for genes in WT and MED12 knockout cell lines. Datapoints are colored by LMNB1 DamID score. (**F**) Correlation between genic LMNB1 DamID score in WT and H3K9me3 levels in WT cells. The blue line (**E**, **F**) represents a fitted linear model; Pearson correlation and *P* values are shown in the plots. Results (**E**, **F**) are from three biological replicates. Data are from (El Khattabi et al, 2019; Leemans et al, 2019; Lidschreiber et al, 2021; Schick et al, 2021; Haarhuis et al, 2022, 35136067). For (**A**–**C**), the central line in the boxplots represents the median. The lower and upper hinges correspond to the first and third quartiles (the 25th and 75th percentiles). The upper and lower whiskers extends from the hinge to the largest or smallest values respectively no further than 1.5 * inter-quartile range. Outliers were removed only for visualization purposes.

 © The Author(s)
