# Supplementary material for: Chromatin protein complexes involved in gene repression in lamina-associated domains
Source: EMBO J. 2024 Sep 25;43(21):5260–87. doi: 10.1038/s44318-024-00214-1 (PMC11535540; doi:10.1038/s44318-024-00214-1)
Supplement: Supplementary file 4 — EV and Appendix Figure Source Data [file 44318_2024_214_MOESM4_ESM.zip › Appendix Figure Source Data.zip!/EMBOJ_2024_117408_RawData_AppendixFigureS1A.pdf]

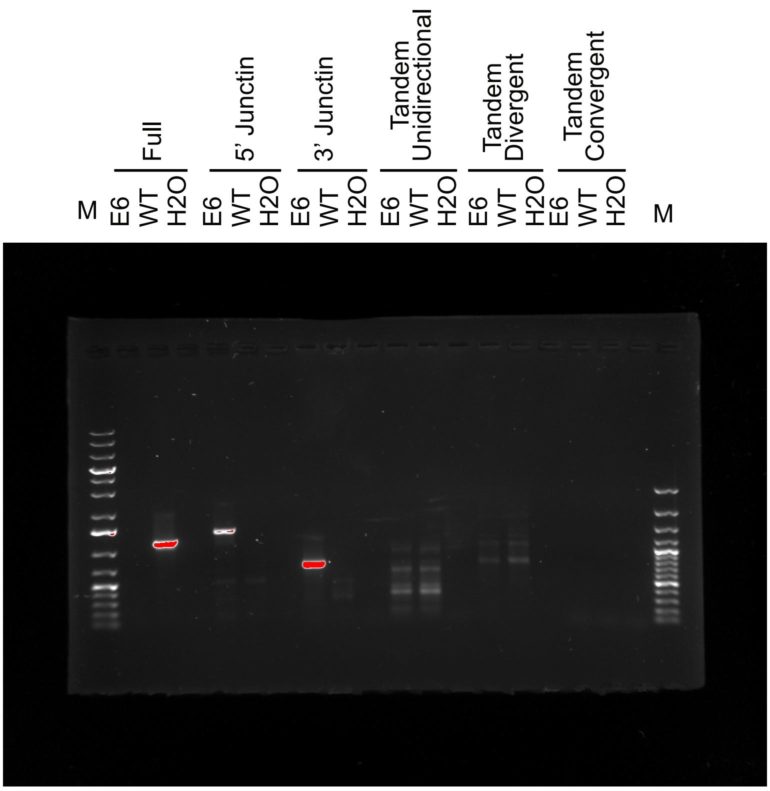
M
E6 WT H2O
Full
E6 WT H2O
5' Junctin
E6 WT H2O
3' Junctin
E6 WT H2O
Tandem Unidirectional
E6 WT H2O
Tandem Divergent
E6 WT H2O
Tandem Convergent
M

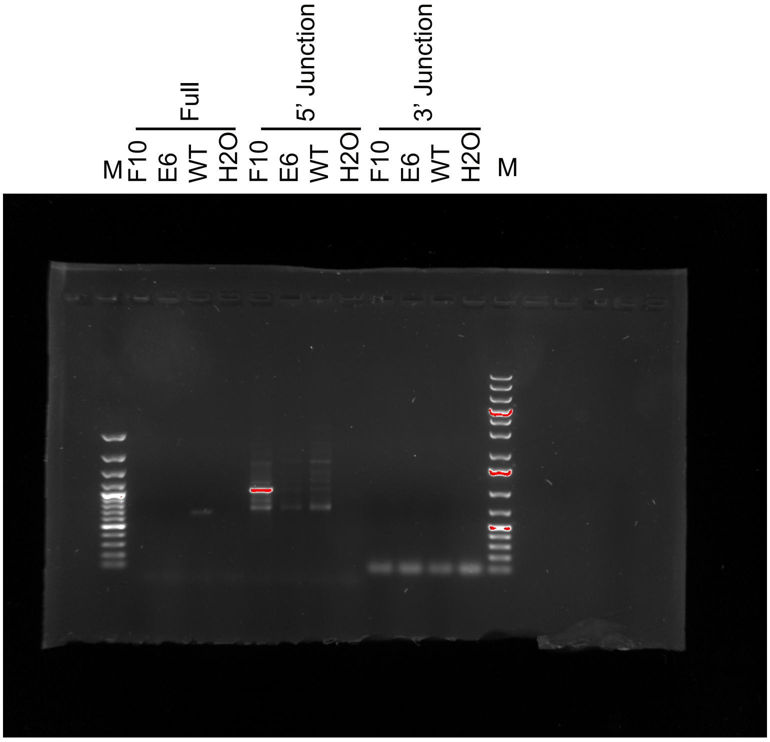
M
F10 E6 WT H2O
Full
F10 E6 WT H2O
5' Junction
F10 E6 WT H2O
3' Junction
M

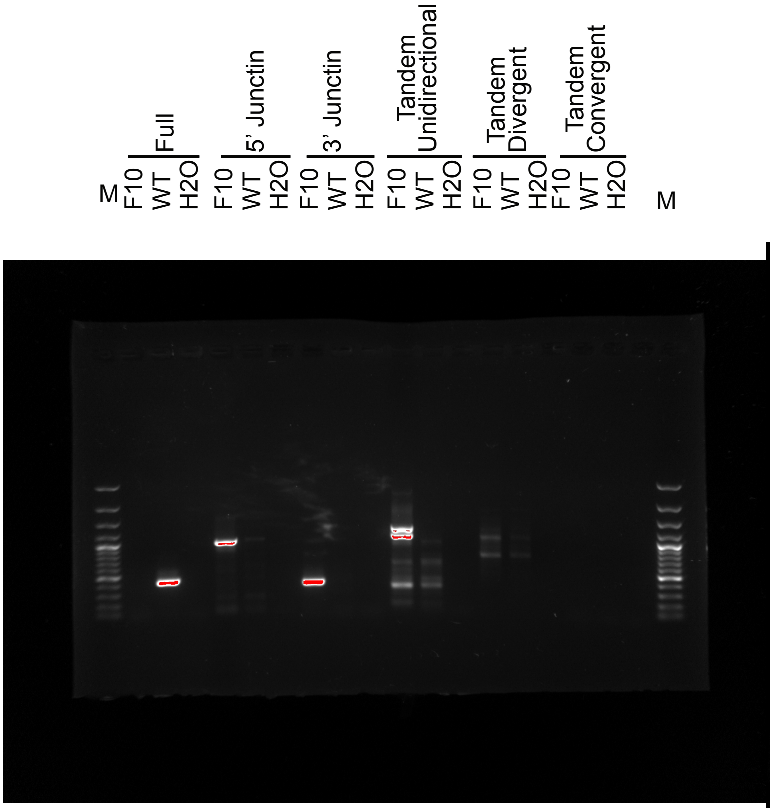
M
F10 WT H2O
Full
F10 WT H2O
5' Junctin
F10 WT H2O
3' Junctin
F10 WT H2O
Tandem Unidirectional
F10 WT H2O
Tandem Divergent
F10 WT H2O
Tandem Convergent
M

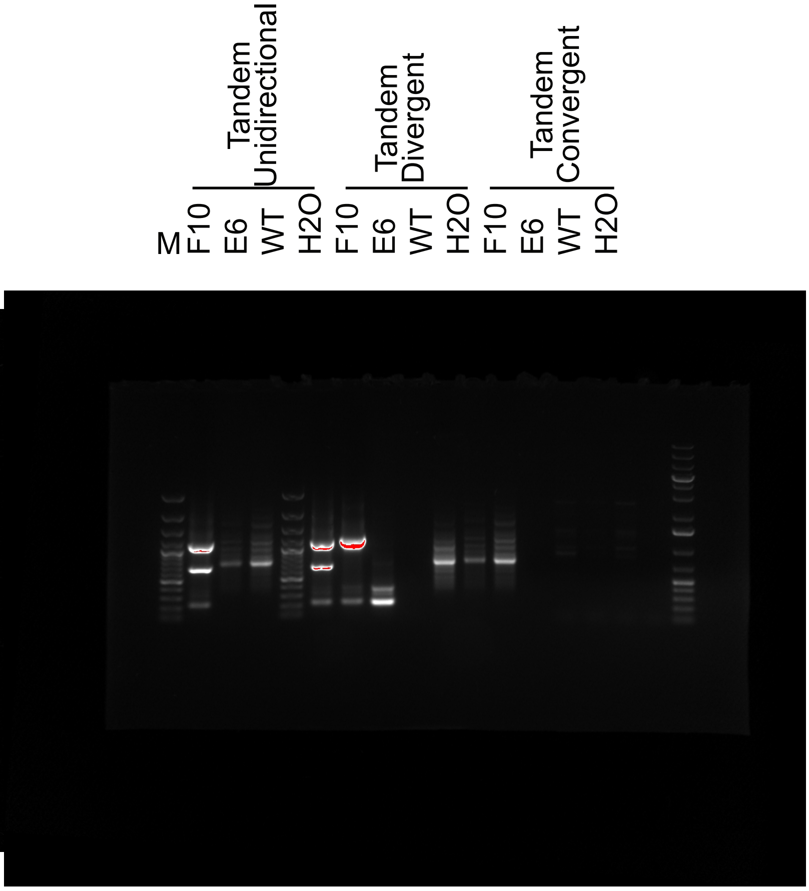
M
F10 E6 WT H2O
Tandem Unidirectional
F10 E6 WT H2O
Tandem Divergent
F10 E6 WT H2O
Tandem Convergent
