# Supplementary material for: Chromatin protein complexes involved in gene repression in lamina-associated domains
Source: EMBO J. 2024 Sep 25;43(21):5260–87. doi: 10.1038/s44318-024-00214-1 (PMC11535540; doi:10.1038/s44318-024-00214-1)
Supplement: Supplementary file 4 — EV and Appendix Figure Source Data [file 44318_2024_214_MOESM4_ESM.zip › EV Figure Source Data.zip!/EMBOJ_2024_117408_RawData_FigEV4A.pdf]

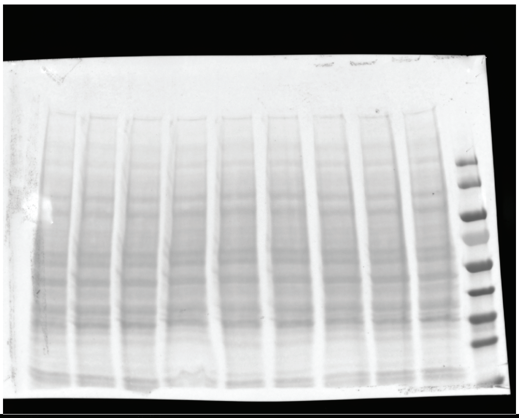

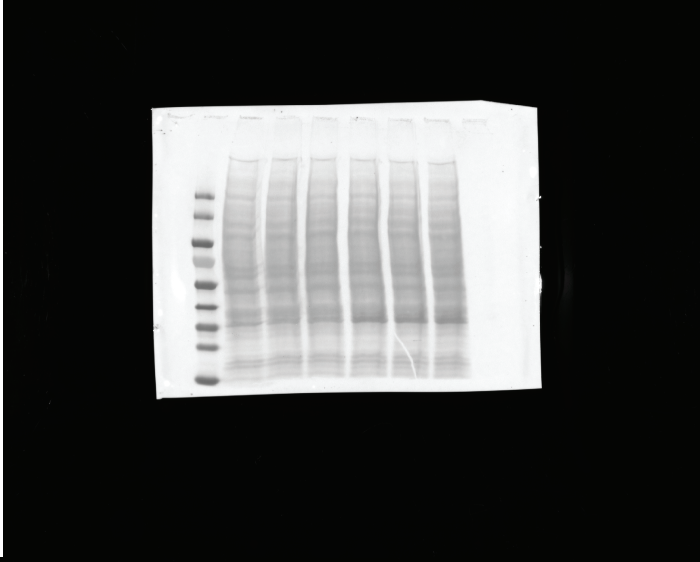

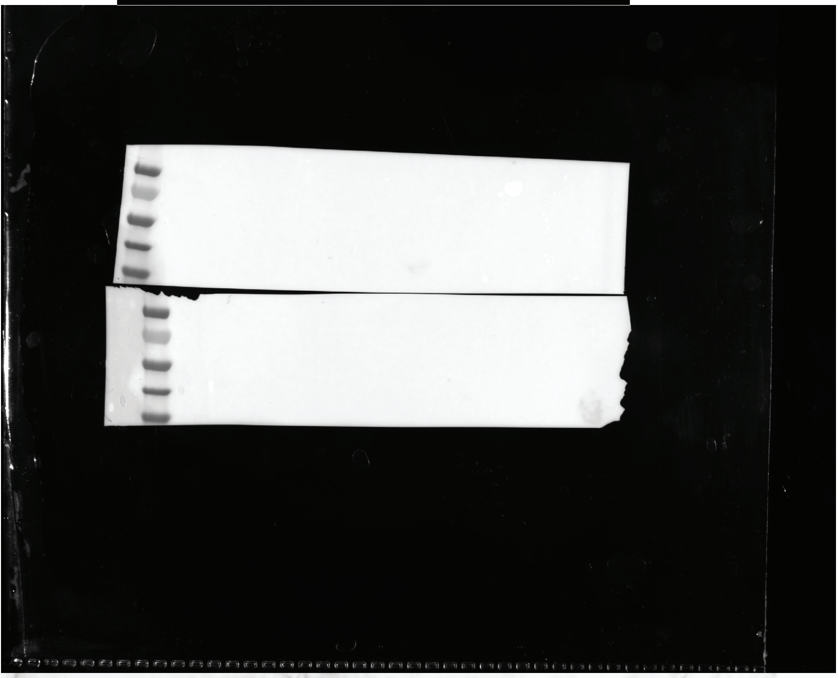

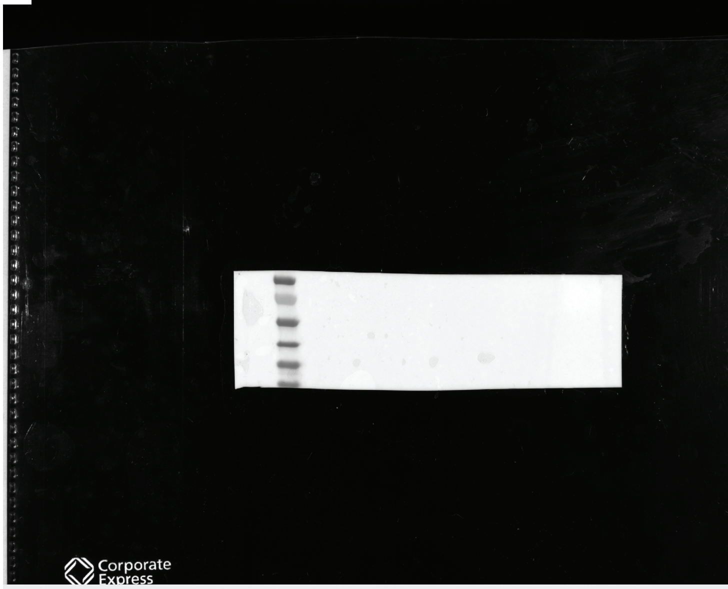
Corporate Express

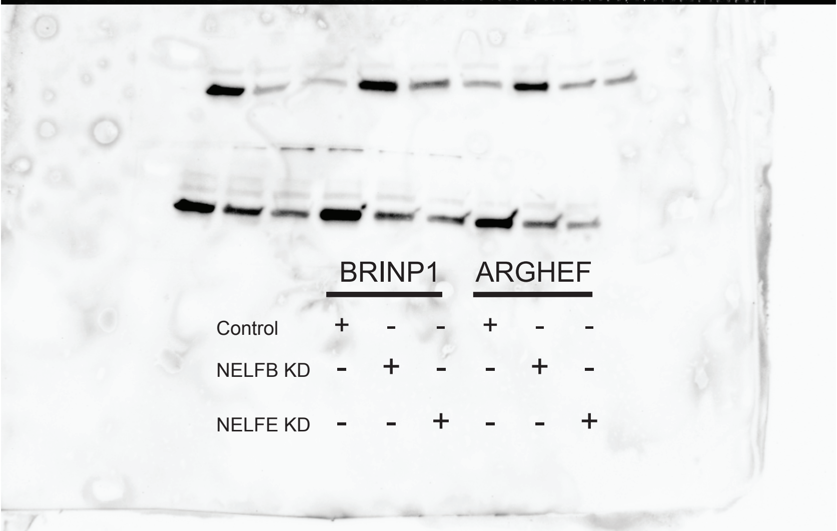
BRINP1
ARGHEF
Control + - - + - -
NELFB KD - + - - + -
NELFE KD - - + - - +

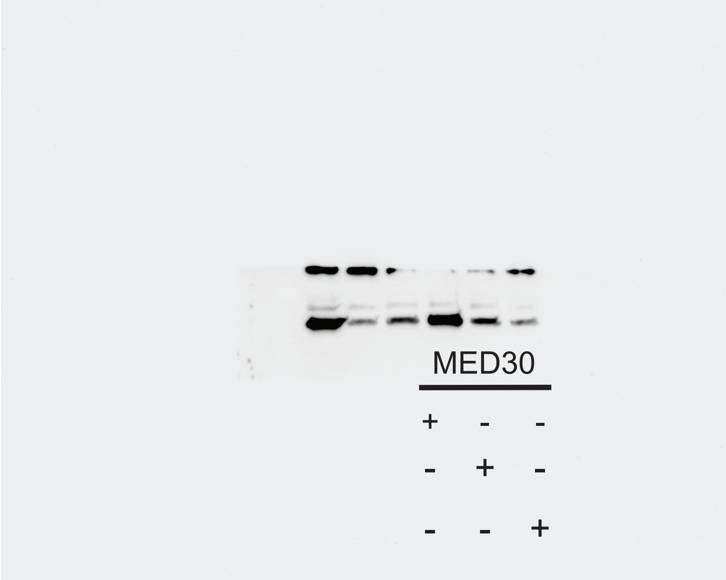
MED30
+ - -
- + -
- - +
